# Influence of laser powder bed fusion build orientation on the corrosion resistance of CoCrWMo alloy considering dislocation density character and lattice microstrain

**DOI:** 10.1038/s41598-025-30143-w

**Published:** 2025-11-28

**Authors:** Alicja Stanisławska, Tomasz Seramak, Katarzyna Zasińska, Łukasz Gaweł, Grzegorz Gajowiec, Maria Gazda, Dorota Moszczyńska, Jarosław Mizera, Marek Szkodo

**Affiliations:** 1 Faculty of Mechanical Engineering and Ship Technology, Gdansk University of Technology, Narutowicza 11/12, Gdańsk, 80–233 Poland; 2 Faculty of Chemistry, Gdansk University of Technology, Narutowicza 11/12, Gdańsk, 80–233 Poland; 3 Faculty of Applied Physics and Mathematics, Gdansk University of Technology, Narutowicza 11/12, Gdańsk, 80–233 Poland; 4 Faculty of Materials Science and Engineering, Warsaw University of Technology, Wołoska 141, Warsaw, 02-507 Poland

**Keywords:** Laser powder bed fusion (L-PBF), CoCrWMo alloy, Corrosion resistance, Dislocation density, Lattice microstrain, Engineering, Materials science

## Abstract

This study investigates the impact of build orientation on the corrosion resistance of CoCrWMo alloys fabricated by Laser Powder Bed Fusion (L-PBF). Samples printed at angles from 0° to 90° were characterized using SEM, XRD (with modified Williamson-Hall and Warren-Averbach analysis), and potentiodynamic polarization in Ringer’s solution. The results revealed that build orientation strongly affects microstrain, dislocation density, and dislocation character, which in turn govern passive layer composition and corrosion behavior. The sample printed at 90° exhibited the highest corrosion resistance, correlating with the lowest dislocation density, minimal microstrain, and the highest fraction of screw dislocations. XPS analysis showed this sample had the most Cr-rich passive layer, with Cr₂O₃ accounting for over 60% of oxide species. In contrast, low-angle samples exhibited more edge dislocations, higher microstrain, and less protective oxide films. These findings highlight the critical role of build-induced crystallographic anisotropy and defect structure in shaping corrosion response in L-PBF-processed biomedical alloys. The study also demonstrates the utility of advanced XRD-based methods for quantifying lattice distortions and dislocation populations. Importantly, the results support sustainable design strategies by enhancing corrosion resistance through process control, reducing reliance on coatings or alloying, and promoting resource-efficient manufacturing of biomedical and engineering components.

**Supplementary Information:**

The online version contains supplementary material available at 10.1038/s41598-025-30143-w.

## Introduction

Laser Powder Bed Fusion (L-PBF) is one of the most widely adopted additive manufacturing (AM) techniques for the production of high-performance metal components, particularly in the biomedical and aerospace sectors^1–3^. Among the materials suitable for L-PBF AM processing, CoCr-based alloys have gained significant attention due to their excellent wear resistance, high corrosion resistance, and mechanical strength, making them ideal for load-bearing implants and tribological components^4–6^.

During the L-PBF process, the build orientation significantly affects the local thermal gradients and solidification conditions, thereby influencing grain morphology, crystallographic texture, dislocation structure, and residual stress distribution^7–9^. It has been shown that build angle variation may lead to changes in columnar grain alignment, phase stability, and substructure development, which ultimately alter the mechanical and functional properties of the printed alloy^10–12^. Importantly, the ability to tailor corrosion resistance through process parameter selection aligns with the principles of sustainable manufacturing, enabling performance optimization without additional material inputs, coatings, or environmentally burdensome treatments. Despite numerous studies addressing the mechanical behavior and microstructure of CoCrMo alloys fabricated by L-PBF, there is limited understanding of how build orientation affects their corrosion resistance, especially in relation to the underlying crystallographic distortions and dislocation characteristics.

Previous research has demonstrated that dislocation density and type play a crucial role in determining the passive layer formation and stability in Co-based alloys^13–15^. Dislocations can act as fast diffusion paths for ionic species and can also influence the nucleation of oxides, altering the thickness, composition, and protectiveness of the passive film^16,17^. However, most corrosion studies focus on surface roughness, porosity, or alloying effects, without detailed microstructural correlations, particularly regarding strain-induced lattice distortion and dislocation character.

The impact of L-PBF build orientation on strain accommodation and dislocation behavior has been investigated primarily in the context of mechanical performance^18–20^, with limited work linking these features to electrochemical properties. Some studies have suggested that microstructural anisotropy introduced by laser scanning strategies may also affect electrochemical stability by influencing grain boundary distribution and passive layer uniformity^21,22^. Yet, a comprehensive study correlating build angle, dislocation mechanics, and passive layer chemistry is still lacking.

To bridge this knowledge gap, this study investigates the effect of L-PBF build orientation on the corrosion resistance of a CoCrWMo alloy in relation to crystallographic distortion and dislocation behavior. A wide range of build angles (0° to 90°) was used to induce varying grain orientations and cooling rates. X-ray diffraction (XRD) analysis, including a modified Williamson–Hall approach, was employed to quantify microstrain, crystallite size, dislocation density, and the relative contributions of screw and edge dislocations. The chemical composition of the passive layers was assessed using X-ray photoelectron spectroscopy (XPS), providing insights into oxide formation as a function of internal strain and defect structure.

The novelty of this study lies in the quantitative link established between build-induced microstructural anisotropy, dislocation character, and the composition of passive films that govern corrosion resistance. By integrating advanced XRD analysis with electrochemical evaluation and surface chemistry characterization, this work contributes to the understanding of structure–property relationships in additively manufactured CoCr alloys. The findings provide a scientific basis for optimizing build parameters to enhance corrosion performance in biomedical and functional applications.

## Materials and methods

### Powder bed fusion additive manufacturing

Samples used for the study were fabricated via the L-PBF technique using gas-atomized Co-Cr alloy powder supplied by S&S Scheftner GmbH (Mainz, Germany). The powder, designed for permanent and removable dental restorations, complied with ISO 22,674 (type 5) and was free of nickel, cadmium, beryllium, and lead. Chemical composition is below in Table 1.

**Table 1:** Chemical composition of printed alloy.

Compound	Co	Cr	Wo	Mo	Si	Fe	Mg	C, *N*
Wt.%	59.93	25.71	9.57	2.35	0.92	0.25	0.02	< 1

**Fig. 1:**
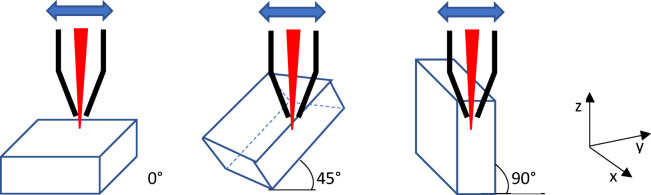
Schematic illustration of sample orientation during powder bed fusion additive manufacturing (L-PBF) at build angles of 0°, 45°, and 90° relative to the substrate. The laser beam movement is indicated by the double-headed arrow, and the laser incidence direction is shown in red. The xyz coordinate system defines the spatial reference for orientation.

A particle size under 50 μm was used. The components were produced using a Realizer L-PBF 100 machine. Laser power settings ranged from 45 to 55 W for the outer contour and 55–75 W for the inner fill, with a laser spot diameter of 0.13 mm. The layer thickness was 25 μm, and the process was carried out under an argon atmosphere with oxygen concentration maintained at 0.2%. The Co-Cr base plate was heated to 200 °C. Cuboidal samples (10 × 10 × 5 mm) were fabricated at various build angles from 0° to 90°, in 15° increments, as illustrated in Fig. 1. After printing, the supports were removed, and the samples were sandblasted.

Prior to corrosion testing, all samples were ultrasonically cleaned in ethanol and deionized water, dried in warm air, and mechanically polished using SiC abrasive papers up to #4000 grit, followed by final polishing with 0.05 μm alumina suspension to achieve a mirror-like finish. This preparation ensured comparable surface roughness across all specimens and eliminated the influence of surface irregularities originating from the additive manufacturing process. The polished samples were then rinsed again in deionized water and stored in a desiccator until testing.

### Electrochemical measurements and data analysis

Electrochemical tests were conducted to assess the corrosion resistance and passive film stability of the samples. A conventional three-electrode setup was employed, with the sample as the working electrode, Ag/AgCl as the reference, and a platinum mesh as the counter electrode.

The corrosion tests were performed in Ringer’s solution prepared using Ringer tablets (Millipore Sigma–Aldrich, product no. 115525). Each tablet was dissolved in 500 mL of distilled water, according to the manufacturer’s instructions. The resulting electrolyte contained approximately 1.125 g NaCl, 0.0525 g KCl, 0.03 g CaCl₂, and 0.025 g NaHCO₃ per 500 mL, yielding a pH of 6.8–7.2 at 25 °C, consistent with physiological conditions. All tests were performed at room temperature (23 ± 1 °C) under naturally aerated conditions.

Open circuit potential (OCP) was monitored for 3600 s under static conditions. The stabilized OCP was defined as the average over the final 300 s. Higher and more stable OCP values were associated with rapid and uniform passive film formation, while lower or unstable readings indicated delayed passivation or surface heterogeneity.

Following OCP stabilization, potentiodynamic polarization curves were recorded from − 1.0 V to + 1.0 V (vs. Ag/AgCl) at a scan rate of 1 mV/s. Corrosion potential (Ecorr) and current density (i_corr_) were determined as the potential and current near the minimum net current at the cathodic-anodic transition. Classical Tafel extrapolation was not applied due to the absence of linear regions in most cases. This behavior is typical for Co–Cr–based alloys in Ringer’s solution, where rapid passivation and local film breakdown occur shortly after polarization begins. As a result, the current density deviates from linearity even within ± 150 mV around Ecorr, making Tafel fitting unreliable. Preliminary fitting attempts yielded poor correlation coefficients (R² < 0.90), confirming the lack of well-defined Tafel slopes (β_a and β_c).

Passive current density (i_pass_) was calculated as the mean logarithmic current within the − 0.4 V to + 0.2 V range. Breakdown potential (E_bd_) was defined as the potential at which anodic current increased by at least one order of magnitude, indicating passive film failure.

All data were processed in Python, with parameter validation via visual inspection of polarization curves.

### Material characterization

#### X-ray diffraction line profile analysis using the Warren-Averbach method

The Warren-Averbach (W-A) method was employed to quantify microstrain and assess the potential contribution of crystallite size in L-PBF-processed CoCrWMo alloy. XRD patterns were collected using a Philips X’Pert Pro diffractometer (Co Kα, λ = 0.15405 nm) in Bragg-Brentano geometry over a 2θ range of 20°−110° with a step size of 0.002°.

Raw data were smoothed, background-corrected, and converted to reciprocal space using g = 2sinθ/λ. Selected peaks were extracted symmetrically, normalized to unit intensity, and centered to g_0_ = 0. The cosine Fourier transform of the normalized intensity I′(g_i_) was computed for Fourier lengths L ∈^1,5^ nm:

1 $$\:A\left(L\right)=\varDelta\:g\cdot\:\sum\:{I}^{{\prime\:}}\left({g}_{i}\right)\cdot\:\mathrm{c}\mathrm{o}\mathrm{s}\left[2\pi\:\left({g}_{i}-{g}_{0}\right)L\right]$$

W-A analysis relies on^23,24^:

2 $$\:lnA\left(L\right)=-\frac{L}{D}-2{\pi\:}^{2}{\epsilon\:}^{2}{g}_{0}^{2}{L}^{2}$$

For small L, the linear term is negligible and microstrain is estimated from the slope k of the lnA(L) vs. L^2^ plot as:

3 $$\:\epsilon\:=\sqrt{\frac{-k}{2{\pi\:}^{2}{g}_{0}^{2}}}$$

For large L, the decay should be dominated by crystallite size:

4 $$\:lnA\left(L\right)\approx\:-\frac{L}{D}$$

However, in all samples, nonphysical increasing trends were observed in the lnA(L) vs. 1/L plots, indicating that lattice microstrain dominated peak broadening. Consequently, only ε values from the W-A method were considered reliable.

Crystallite size D was instead estimated using the Scherrer Eqs^25–27^.:

5 $$\:D=\frac{{a}_{s}}{{\beta\:}_{g}}$$

where a_s_ = 0.9 (typical for columnar, textured grains in L-PBF alloys), and β_g_ ​ is the full width at half maximum (FWHM) in reciprocal space, obtained by Lorentzian fitting of the (111) peak. Instrumental broadening was corrected using a standard Si powder (NIST SRM 640 d) measured under identical conditions.

#### Dislocation character

The fraction of screw and edge dislocations in the L-PBF-fabricated CoCrWMo alloy was determined using the lever rule:

6 $$\:{f}_{screw}=\frac{q-{q}_{edge}^{th}}{{q}_{screw}^{th}-{q}_{edge}^{th}}=1-{f}_{edge}$$

Here, q is the experimental parameter obtained from modified Williamson-Hall (mW-H) analysis, and q^th^_screw_, q^th^_edge_ ​ are theoretical reference values assuming 100% screw or 100% edge dislocations, respectively.

The theoretical q^th^ values were calculated using the empirical relation:

7 $$\:{q}_{i}^{th}={a}_{i}^{q}\left[1-exp\left(\frac{-A}{{b}_{i}^{q}}\right)\right]+{A\cdot\:c}_{i}^{q}+{d}_{i}^{q}$$

where A = 2C_44_/(C_11_ − C_12_) is the elastic anisotropy coefficient, and a_i_^q^, b_i_^q^, c_i_^q^, d_i_^q^ ​ are dislocation-type-specific constants obtained from literature^28–30^. For Co-based FCC alloys (referencing CoCrFeNi), the elastic constants were taken as: C_11_ = 221 GPa, C_12_ = 131.8 GPa, C_44_ = 129 GPa, yielding A = 4.01^31,32^.

The experimental q parameter was determined from the linearized form of the mWH method developed by Ungár et al.^29^:

8 $$\:{\left(\frac{\varDelta\:K-\alpha\:}{K}\right)}^{2}={\beta\:}^{2}\cdot\:\stackrel{-}{C}\left({1-qH}^{2}\right)$$

where ΔK is the integral breadth in reciprocal space, K = 2sinθ/λ, and α = a_s_/D accounts for size-related broadening. The dislocation parameter β is defined as:

9 $$\:\beta\:=bM\sqrt{\frac{\pi\:\rho\:}{2}}$$

where b is the Burgers vector, M is a constant related to elastic anisotropy (typically M = 2.75 for FCC structures), and ρ is the dislocation density.

The dislocation contrast factor C̅ is calculated separately for screw and edge dislocations, and H^2^ is the orientation factor:

10 $$\:{H}^{2}=\frac{{h}^{2}{k}^{2}+{k}^{2}{l}^{2}+{h}^{2}{l}^{2}}{{\left({h}^{2}+{k}^{2}+{l}^{2}\right)}^{2}}$$

A linear fit of (ΔK − α)^2^/K^2^ versus H^2^ yields the parameter q from the intersection point with the H^2^ axis (i.e., 1/q). The relative contributions of screw and edge dislocations are then calculated using Eq. (6) based on the theoretical bounds from Eq. (7).

#### Dislocation density determination

The total dislocation density (ρ) was determined using the mW-H approach, which relates the peak broadening ΔK in reciprocal space to both crystallite size and dislocation density^33^:

11 $$\:\varDelta\:K\cong\:\frac{{a}_{s}}{D}+bM\sqrt{\frac{\pi\:}{2}\rho\:\left(K\cdot\:{\stackrel{-}{C}}^{\raisebox{1ex}{$1$}\!\left/\:\!\raisebox{-1ex}{$2$}\right.}\right)}$$

where *D* is the crystallite size, *b* is the Burgers vector, *M* is a dimensionless constant (typically 2.75 for FCC materials), *K* is the scattering vector, and C̅ is the dislocation contrast factor.

The contrast factor C̅ is calculated as:

12 $$\:\stackrel{-}{C}={\stackrel{-}{C}}_{h00}\left(1-q{H}^{2}\right)$$

Here, C̅_h00_ is the base contrast factor for the (h00) reflection, and *H²* is the orientation factor defined by the Miller indices (see Eq. 10).

The theoretical C̅_h00_ values for edge and screw dislocations were calculated using:

13 $$\:{\stackrel{-}{C}}_{{h00}_{i}}={a}_{i}^{{C}_{h00}}\left[1-exp\left(\frac{-A}{{b}_{i}^{{C}_{h00}}}\right)\right]+A\cdot\:{c}_{i}^{{C}_{h00}}+{d}_{i}^{{C}_{h00}}$$

where *A* is the elastic anisotropy coefficient, and the coefficients a_i_^Ch00^, b_i_^Ch00^, c_i_^Ch00^, d_i_^Ch00^ depend on dislocation character (edge or screw) and slip system geometry. The final C̅_h00_ value was calculated as a weighted average based on the relative fractions of screw and edge dislocations obtained using Eq. (7).

Once C̅ was computed, a linear fit of ΔK vs. K⋅C̅ ^1/2^ enabled determination of dislocation density ρ from the slope, as per Eq. (11).

### Chemical composition of the passive layer - XPS analysis

X-ray photoelectron spectroscopy (XPS) was performed using a ThermoFisher Scientific Escalab 250Xi system with a monochromatic Al Kα source. High-resolution spectra were acquired in Constant Analyzer Energy (CAE) mode at 20.0 eV pass energy, with a step size of 0.100 eV (181 energy steps) and a spot size of 500 μm. A total of 25 scans were collected per spectrum, with an acquisition time of 3 min 46.2 s.

The spectra were calibrated to the C 1 s peak at 284.8 eV corresponding to adventitious carbon. Peak fitting and quantitative analysis were performed using Thermo Avantage software (Thermo Fisher Scientific), employing a Shirley background and mixed Gaussian-Lorentzian peak shapes (GL(30)).

Quantitative analysis involved deconvolution of high-resolution spectra into distinct chemical states (metallic, oxide, carbide), followed by integration of peak areas and correction using element-specific relative sensitivity factors (RSFs). Atomic percentages (at%) were calculated as:

14 $$\:at.\mathrm{\%}=\left(\frac{{I}_{corrected}}{\sum\:{I}_{corrected}}\right)\cdot\:100\mathrm{\%}$$

where I_corrected_ denotes the RSF-adjusted intensity for each species.

The analysis focused on variations in surface chemistry across samples fabricated at different build angles, emphasizing both elemental content and oxidation states. Particular attention was given to protective oxides, such as Cr₂O₃, due to their critical role in corrosion resistance. The findings were interpreted in relation to the electrochemical behavior of the samples.

## Results and discussion

### XRD investigation

**Fig. 2:**
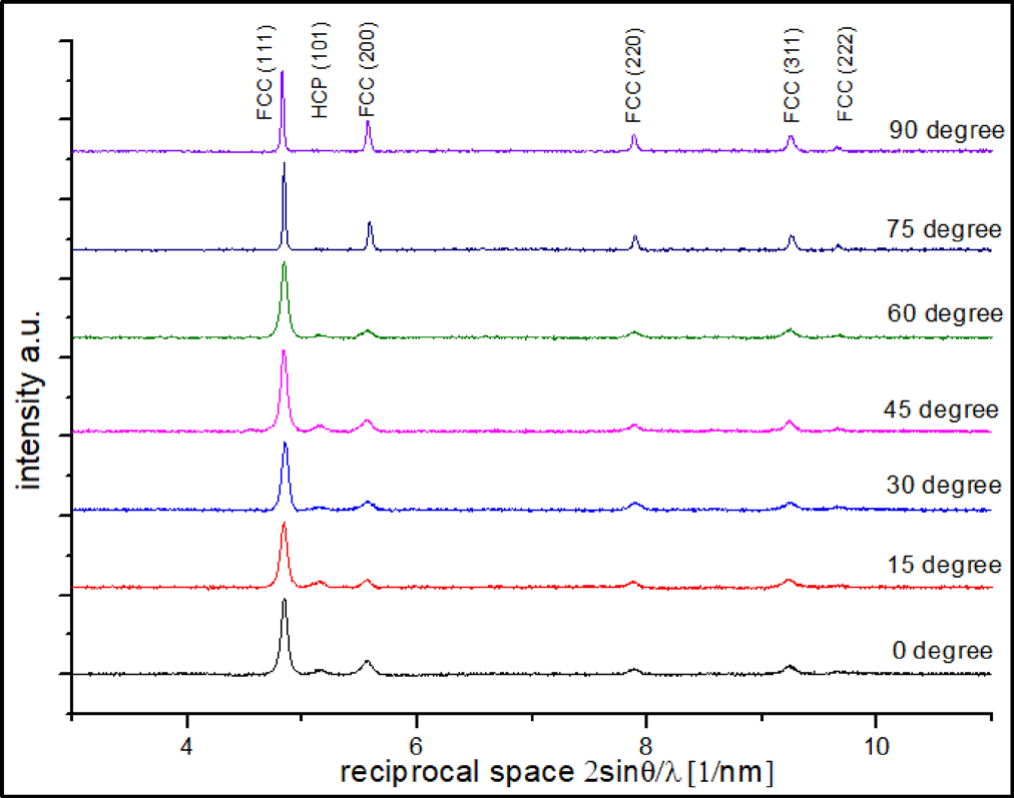
XRD diffraction patterns in reciprocal space (2sinθ/λ) for CoCrWMo alloy samples fabricated by L-PBF at different support angles.

Figure 2 shows the X-ray diffraction patterns in reciprocal space (2sinθ/λ) for CoCrWMo alloy samples fabricated via L-PBF at various build angles (0° to 90°). All samples exhibit sharp and distinct peaks corresponding to the face-centered cubic (FCC) phase, including the (111), (200), (220), (311), and (222) reflections. This confirms the dominant FCC structure across all orientations.

Notably, for samples built at lower angles (0°–45°), an additional peak appears at ≈ 5.1 nm⁻¹, attributed to the (101) plane of the hexagonal close-packed (HCP) phase. This suggests partial FCC→HCP transformation under reduced cooling rates typical for low-angle orientations. The finding aligns with known solidification behavior in cobalt-based alloys, where HCP formation is favored at slower cooling rates due to thermodynamic stability and transformation kinetics^34^.

At higher build angles, this HCP-related peak diminishes or disappears entirely, indicating suppression of the transformation and full retention of the metastable FCC phase due to faster cooling. These phase and texture variations will be further analyzed in the context of peak broadening and Fourier-based line profile analysis in subsequent sections.

### Electrochemical measurements

The electrochemical results are presented prior to the microstructural analysis to first establish the corrosion performance trends, which are subsequently interpreted in terms of surface morphology and phase composition.

**Fig. 3:**
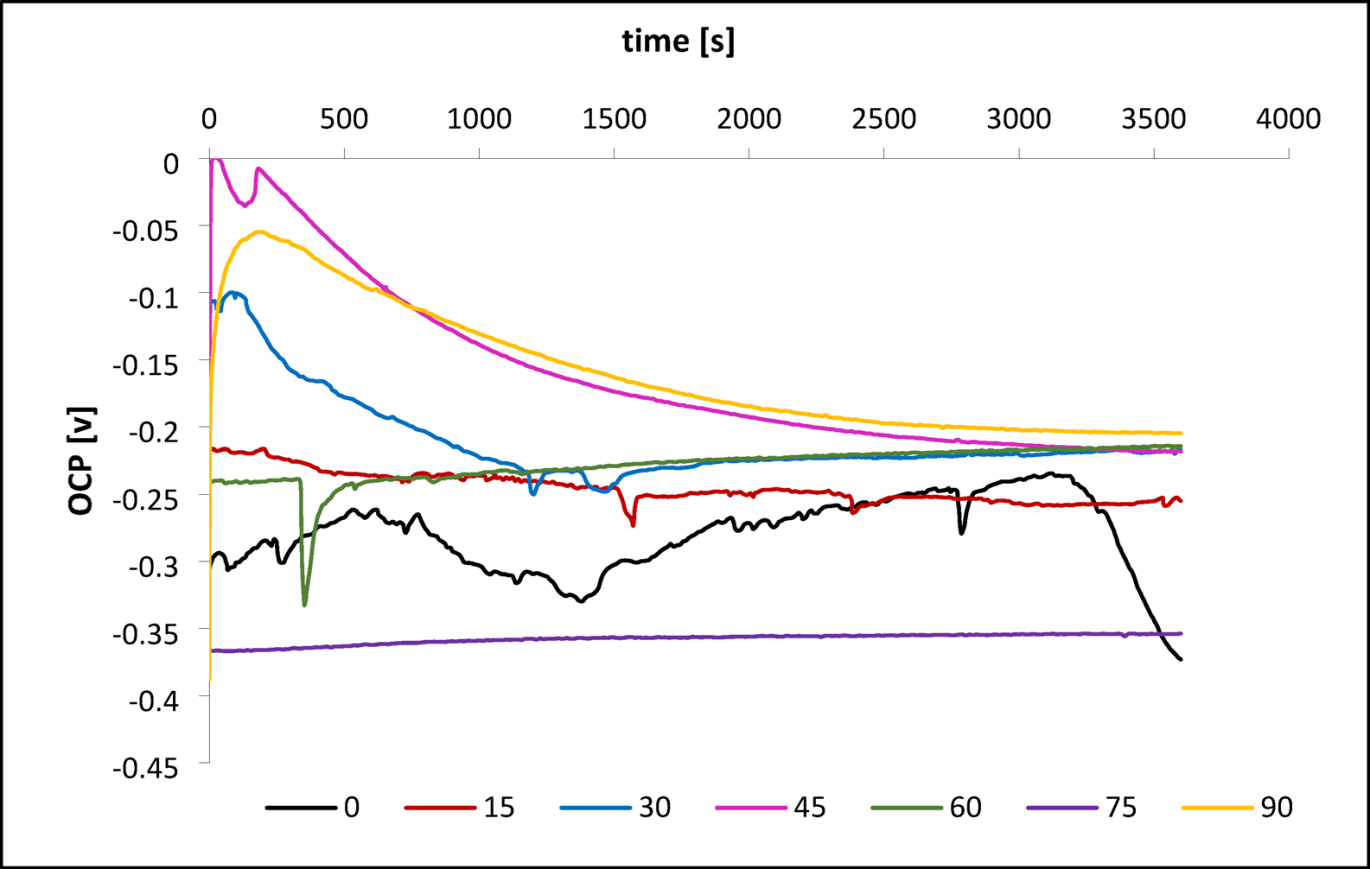
Open circuit potential (OCP) curves of CoCrWMo alloy samples fabricated by L-PBF at different support angles (0°−90°) during immersion in Ringer’s solution.

Figure 3 shows the evolution of open circuit potential (OCP) for L-PBF-fabricated CoCrWMo samples at different build angles in Ringer’s solution. OCP measurements reflect early electrochemical stability and passive film formation dynamics.

Samples fabricated at low build angles (0° and 15°) exhibited unstable electrochemical behavior. The 0° sample showed pronounced potential fluctuations with sharp negative shifts and incomplete recovery, stabilizing at ~- 0.37 V. The 15° sample displayed a gradual decline with minor transients, reaching ~- 0.257 V. These features suggest delayed or locally unstable passivation, likely due to heterogeneous surface structures and high dislocation densities.

In contrast, intermediate-angle samples (30°, 45°, 60°) demonstrated improved passivation. The 30° sample stabilized at ~- 0.218 V after an initial drop and recovery. The 45° sample exhibited moderate oscillations before reaching ~- 0.255 V, while the 60° sample showed a minor dip followed by steady upward drift, ending near − 0.217 V. These patterns indicate the development of more continuous and protective passive layers.

The 75° and 90° samples displayed the most stable OCP profiles. The 75° sample showed a monotonic potential increase, stabilizing at − 0.354 V without sharp transients. The 90° sample exhibited a rapid early rise to − 0.05 V, followed by a slow decline to a stable − 0.202 V, reflecting rapid surface activation and robust passivation.

Overall, higher build angles promoted more stable OCP behavior and more protective passive films, likely due to refined microstructures and lower defect density. In contrast, lower angles correlated with unstable passivation and inferior electrochemical performance.

#### Potentiodynamic behavior and passivation characteristics

**Fig. 4:**
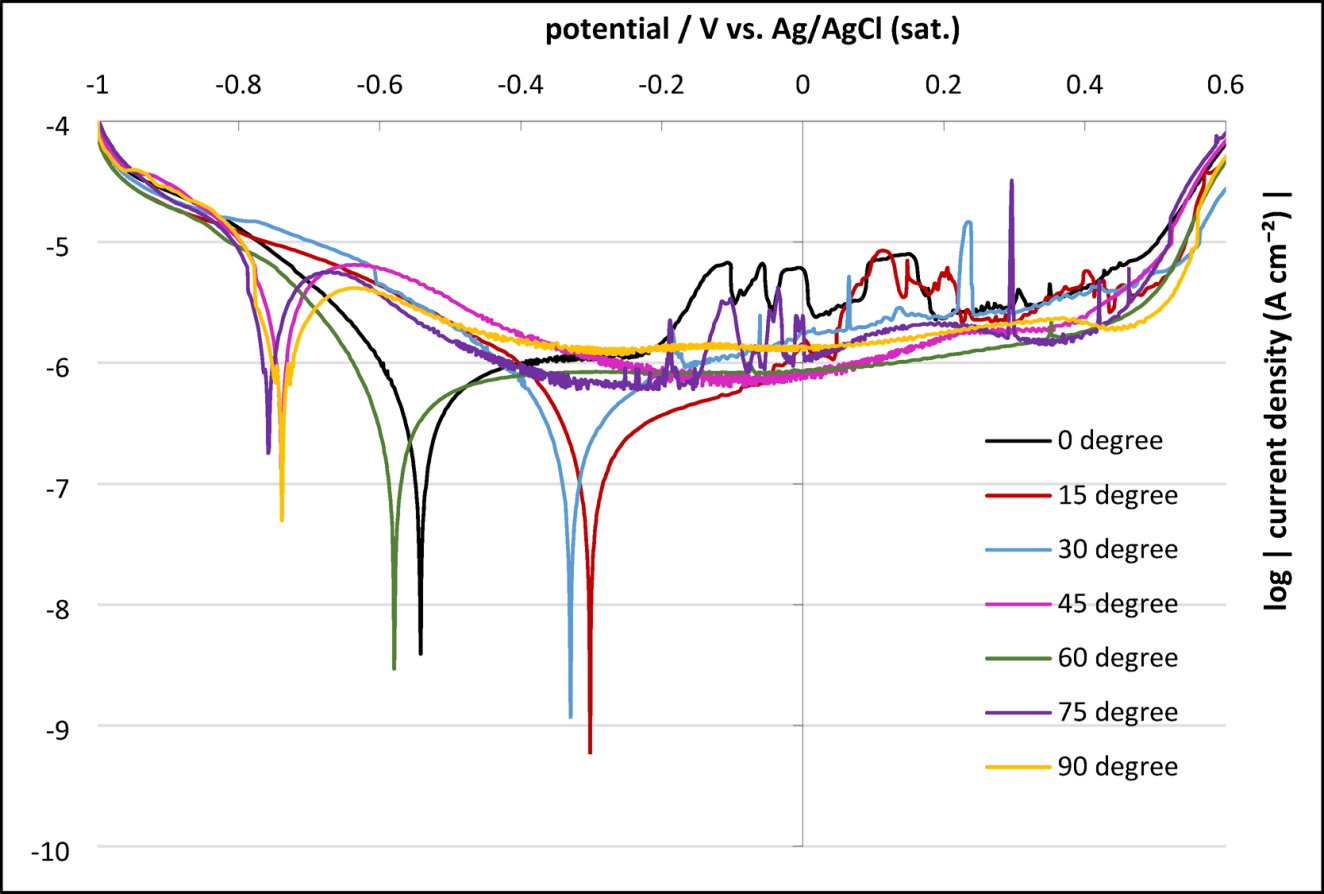
Potentiodynamic polarization curves of CoCrWMo alloy samples fabricated by PBF additive manufacturing at different build angles (0°–90°) in Ringer’s solution.

Figure 4 presents the potentiodynamic polarization curves for L-PBF-fabricated CoCrWMo samples at various build angles in Ringer’s solution. These curves reveal the corrosion susceptibility and passivation characteristics of the alloy surfaces and corroborate the OCP results.

Potentials are reported vs. saturated Ag/AgCl electrode.

The curves demonstrate differences in corrosion susceptibility and passivation behavior depending on the build orientation.

The 0° sample showed moderate corrosion resistance (E_corr_ = −541.8 mV, icorr = 3.93 nA/cm²), but lacked a stable passive region. A steady current increase and frequent metastable fluctuations (−0.25 V to + 0.25 V) indicated unstable passivation and repeated local film breakdown.

The 15° sample exhibited a more positive Ecorr (−302.4 mV) but higher i_corr_ (58.4 nA/cm²), with no defined passive plateau. The gradual current increase and low fluctuation amplitude suggest formation of a thinner, less protective oxide layer.

The 30° sample showed improved corrosion resistance (E_corr_ = −330.2 mV, i_corr_ = 1.18 nA/cm²). Although lacking a distinct passive plateau, the transient anodic spikes were quickly repassivated, indicating dynamic passivation with localized film instability.

The 45° sample demonstrated the highest corrosion activity (E_corr_ = −741 mV, i_corr_ = 1.3 µA/cm²) despite forming a narrow passive region (log i ≈ −6.0 A/cm²). The onset of transpassivity occurred at 0.53 V. Incomplete passivation may result from high dislocation density and microstructural heterogeneity.

The 60° sample displayed moderate corrosion behavior (E_corr_ ≈ −580 mV, i_corr_ ≈ 2.96 nA/cm²) with a narrow passive plateau (−0.3 V to 0 V) and E_bd_ ≈ + 0.48 V, suggesting partial but less stable passivation.

The 75° sample (E_corr_ ≈ −750 mV, i_corr_ ≈ 0.56 µA/cm²) exhibited a pseudo-passive region (−0.6 V to + 0.3 V) with minor transients and E_bd_ ≈ + 0.53 V. While not fully stable, the passive film was relatively protective.

The 90° sample showed the most stable electrochemical behavior (E_corr_ ≈ −738 mV), with a broad passive region (−0.4 V to + 0.1 V), nearly constant current (log i ≈ −5.93 to −5.83 A/cm²), and E_bd_ ≈ + 0.52 V. The absence of spikes suggests the formation of a dense, uniform oxide film associated with a highly textured, low-defect microstructure.

Table 2 summarizes key electrochemical parameters (E_corr_, i_corr_, i_pass_, E_bd_, passive range, and OCP). The results highlight a clear correlation between build orientation and corrosion behavior. High-angle builds (75°−90°) showed enhanced passivation and resistance to localized corrosion, attributed to refined columnar grains and lower dislocation densities. In contrast, low-angle builds (0°−30°) suffered from poor passivation and frequent metastable events. The 45° sample represents an intermediate case with high corrosion activity despite partial passivation.

These findings underscore the importance of build orientation in tailoring electrochemical performance of L-PBF-fabricated CoCrWMo alloys for biomedical applications.

Overall, the corrosion behavior of the CoCrWMo alloy was strongly dependent on the build orientation. Samples fabricated at low build angles exhibited higher defect densities and residual stresses, which promoted heterogeneous passive film formation and unstable electrochemical behavior. In contrast, samples produced at higher build angles showed more homogeneous and refined microstructures, resulting in the formation of stable passive layers characterized by lower current densities and broader passive regions. These results indicate that the build orientation directly affects defect-driven corrosion mechanisms in additively manufactured CoCr alloys. This relationship is further supported by post-corrosion SEM observations presented in Sect. 3.3.

The applied potential range (− 1.0 V to + 1.0 V vs. Ag/AgCl) was selected to evaluate the stability of the passive film under conditions relevant to physiological environments. The test parameters were not intended to induce visible localized corrosion, such as pitting but rather to assess passivation behavior and film integrity. Consequently the observed surface features correspond to mild etching or transient film instability rather than severe corrosion damage. This interpretation is consistent with the moderate current densities obtained and the absence of pronounced transpassive behavior.

### SEM observation

To correlate electrochemical behavior with surface degradation, SEM analyses were performed on CoCrWMo samples fabricated at different build angles after corrosion testing in Ringer’s solution.

Prior to corrosion tests, each surface was also imaged by SEM at matched magnifications to verify that the observed post-exposure features originated from corrosion rather than from SLM surface texture or polishing artefacts.

**Fig. 5:**
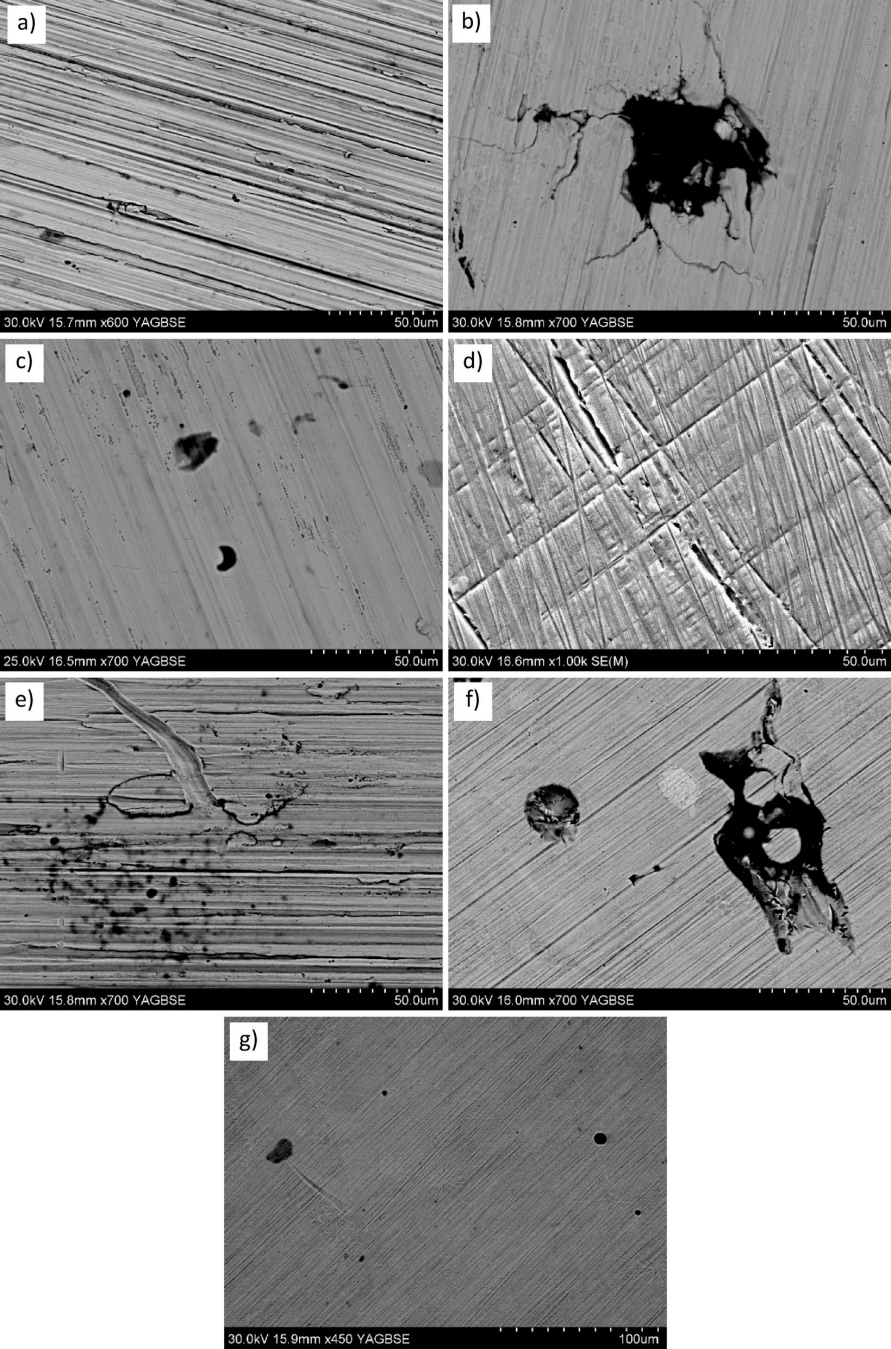
SEM micrographs of the surfaces of CoCrWMo samples after electrochemical corrosion tests in Ringer’s solution, printed at different build angles: (a) 0°, (b) 15°, (c) 30°, (d) 45°, (e) 60°, (f) 75°, and (g) 90°.

It is acknowledged that some surface features observed in Fig. 5(f) may arise from manufacturing related morphology, such as melt-pool boundaries or lack of fusion defects, rather than corrosion attack. Nevertheless, comparative SEM imaging before and after electrochemical testing (see Supplementary Fig. S1) confirms that only shallow etching and minor morphological modifications occurred following exposure. These subtle changes are consistent with mild surface activation rather than deep pitting corrosion, supporting the interpretation that passivation stability, rather than localized breakdown, dominates the observed behavior.

Comparative images before and after testing clearly demonstrate that the as-printed surfaces contained only regular melt-track patterns without pits or cracks, whereas the corroded surfaces exhibit characteristic signs of corrosion damage such as pitting, selective boundary dissolution, and oxide-layer delamination. For completeness, SEM micrographs of the as-printed surfaces before corrosion are provided in the Supplementary Information (Fig. S1) to confirm that the observed post-exposure features originate from corrosion rather than from manufacturing or polishing artefacts.

The SEM images in Fig. 5 reveal distinct patterns of passive film degradation depending on build orientation.

**Table 2:** Summary of electrochemical parameters obtained from open circuit potential (OCP) measurements and potentiodynamic polarization curves for CoCrWMo samples printed at various build angles.

Build angle [°]	Stabilized OCP [V]	E_corr_ [V]	i_corr_ [A/cm²]	passive region [V]	i in passive region [A/cm²]	E_bd_ [V]
**0**	—	- 0.542	3.93 × 10^− 9^	− 0.4 to − 0.21	1.00 × 10^− 6^	+ 0.49
**15**	- 0.257	- 0.302	5.89 × 10^− 10^	—	—	—
**30**	- 0.218	- 0.330	1.17 × 10^− 9^	—	—	—
**45**	- 0.218	- 0.741	9.12 × 10^− 8^	− 0.18 to − 0.16	7.94 × 10^− 7^	+ 0.53
**60**	- 0.217	- 0.580	2.95 × 10^− 9^	− 0.36 to + 0.17	8.51 × 10^− 7^	+ 0.48
**75**	- 0.354	- 0.750	1.78 × 10^− 7^	− 0.31 to − 0.13	6.03 × 10^− 7^	+ 0.44
**90**	- 0.202	- 0.738	5.01 × 10^− 8^	− 0.33 to + 0.13	1.38 × 10^− 6^	+ 0.53

The 0° sample (Fig. 5a) exhibited elongated corrosion grooves along the grinding direction and scattered pits, indicating localized passive film breakdown at surface defects. This suggests corrosion initiated at melt pool boundaries or residual stress concentrations.

The 15° sample (Fig. 5b) showed shallow pits and etched grain boundaries, suggesting partial film degradation and repassivation. A deep pit surrounded by radial cracking implies stress-assisted localized corrosion, consistent with unstable electrochemical behavior.

In the 30° sample (Fig. 5c), isolated pits and shallow cavities were observed, with signs of early-stage degradation or selective dissolution. The absence of large cracks suggests corrosion was localized, supporting the low icorr values but lack of a stable passive plateau.

The 45° sample (Fig. 5d) displayed a mixed morphology with microcracks and corrosion pits, indicating passive layer disruption and possible metastable pitting, in agreement with high icorr and narrow passivation range.

At 60° (Fig. 5e), clustered shallow pits and etched grain boundaries were observed. These features suggest degradation along weaker microstructural regions, consistent with partial passivation and localized transpassive attack.

The 75° sample (Fig. 5f) exhibited a more uniform surface with fine pits and shallow etching. Although signs of localized attack were present, the film appeared capable of repassivation, aligning with the moderate electrochemical stability observed.

Finally, the 90° sample (Fig. 5g) showed minimal corrosion damage, with only a few sub-10 μm pits and a homogeneous background. No signs of film delamination or cracking were detected, confirming the formation of a stable and protective passive layer.

Overall, SEM observations confirm that corrosion resistance improves with build angle, as higher angles promote more uniform microstructures and reduce defect-related passive film breakdown.

### Chemical composition of the passive layer - XPS analysis

**Fig. 6:**
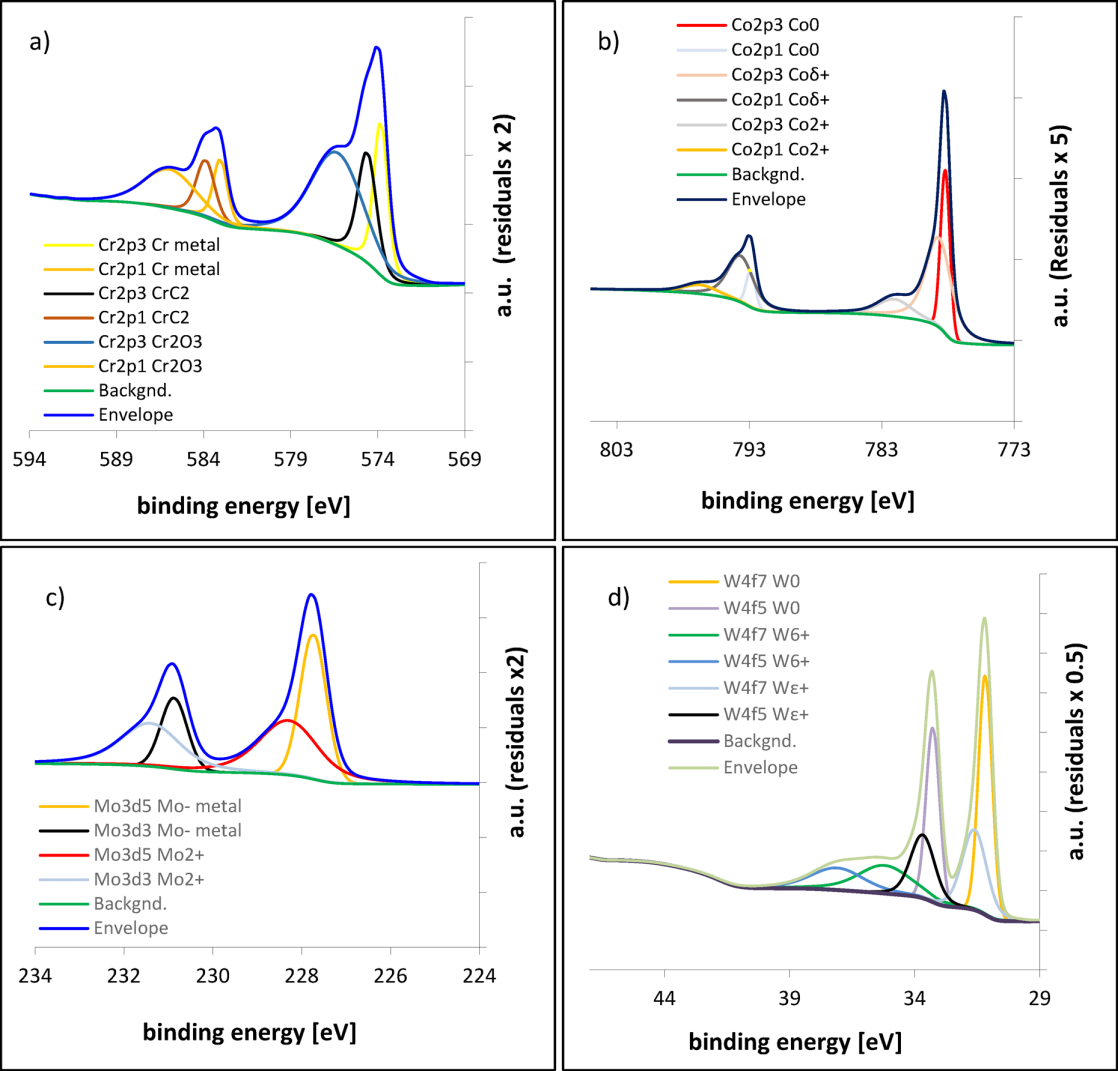
High-resolution XPS spectra of the CoCrWMo sample fabricated at 90° showing the chemical states of: (**a**) chromium (Cr 2p), (**b**) cobalt (Co 2p), (**c**) molybdenum (Mo 3 d), and (**d**) tungsten (W 4f).

**Fig. 7:**
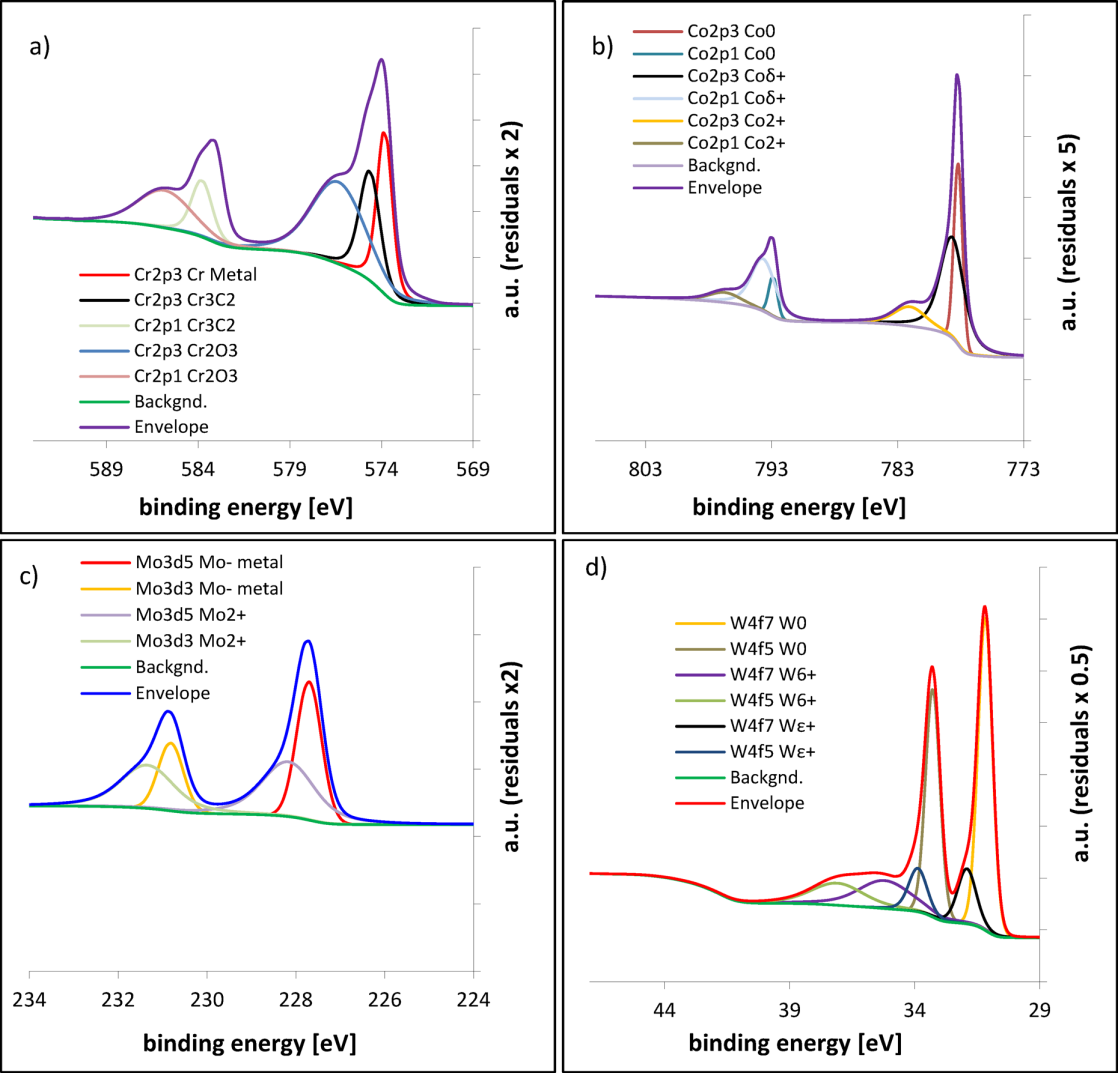
High-resolution XPS spectra of the CoCrWMo sample fabricated at 15° showing the chemical states of: (**a**) chromium (Cr 2p), (**b**) cobalt (Co 2p), (**c**) molybdenum (Mo 3 d), and (**d**) tungsten (W 4f).

XPS analysis was performed after electrochemical testing, ensuring that the detected species correspond exclusively to the passive film. High-resolution XPS spectra for the CoCrWMo alloy samples fabricated at 15° and 90° are shown in Figs. 6 and 7, respectively. The complete quantitative results for all build angles are summarized in Table 3.

A minor Cr₃C₂ component was included in the XPS fitting to account for a small shoulder observed near 574–575 eV, corresponding to carbide related bonding. This feature most likely originates from subsurface carbides formed during solidification and partially exposed after polishing and mild surface etching, rather than from carbide phases present within the passive film itself. The inclusion of this component improves fitting accuracy and allows better separation of overlapping Cr related peaks without implying that carbides are chemically part of the surface oxide layer.

Across all samples, the primary oxides identified include Cr₂O₃ (Cr³⁺), CoO (Co²⁺), MoO₂ (Mo⁴⁺), and WO₃ (W⁶⁺). Among these, Cr₂O₃ dominates the passive layer and plays the decisive role in corrosion protection. The 90° sample, which exhibited the most stable electrochemical behavior, contains over 60% Cr₂O₃ within its total oxide phase. This indicates that the relative enrichment in Cr₂O₃, rather than the overall oxide quantity, governs the effectiveness of passivation.In contrast, samples fabricated at lower build angles (e.g., 15° and 30°) show a more heterogeneous oxide composition, with higher relative fractions of less protective oxides such as WO₃ and MoO₂. Although these oxides contribute to surface passivation, they are more soluble or less stable under physiological conditions, resulting in a more permeable and defect-prone film.Cr₂O₃ is known to form a dense, adherent, and highly stable barrier that underpins corrosion resistance in Cr-bearing alloys. CoO and MoO₂, while present, contribute less effectively due to their porous or semiconducting nature^35,36^. WO₃, although thermodynamically stable, is prone to hydration and leaching, especially in aqueous environments. Therefore, a reduced proportion of WO₃, as observed in the 90° sample, may further enhance the durability of the passive layer.

Overall, the superior corrosion resistance of the 90° sample results not merely from the total oxide content but from the predominance of Cr₂O₃ and the associated microstructural characteristics induced by the build orientation.

Deconvoluted peaks indicate the presence of Cr₂O₃, Cr₃C₂, metallic Cr, CoO, Co²⁺, partially oxidized Co (Co δ⁺), MoO₂, metallic Mo, and various oxidation states of tungsten (W⁶⁺, W⁵⁺, WO₃, and WO). Each fitted component is represented by a distinct color and identified in the legend for clarity.

Deconvoluted peaks indicate the presence of Cr₂O₃, Cr₃C₂, metallic Cr, CoO, Co²⁺, partially oxidized Co (Co δ⁺), MoO₂, metallic Mo, and various oxidation states of tungsten (W⁶⁺, W⁵⁺, WO₃, and WO). Each fitted component is represented by a distinct color and identified in the legend for clarity.

**Table 3:** Surface chemical composition and oxidation States of CoCrWMo samples fabricated at various L-PBF build angles, as determined by XPS analysis. The table includes atomic percentages of metallic and oxidized species, their ratios, and the distribution of individual oxides within the total oxide fraction.

Build angle [°]	Element	Oxidation state	Chemical compound	% at [%]	Metal/metal-oxide ratio	Oxide % within oxides
**0 degree**	Cr	Cr³⁺	Cr₂O₃	26.5	2.2	43.2
	Co	Co²⁺	CoO	13.2	0.84	18.4
	Mo	Mo⁴⁺	MoO₂	10.4	6.2	22.8
	W	W⁶⁺	WO₃	12.6	2.1	15.6
	O	O²⁻	Oxides (total)	48.9	-	-
	C	C-C/C-O	Carbonates/Organics	5.9	-	-
**15 degree**	Cr	Cr³⁺	Cr₂O₃	22.8	1.9	41.2
	Co	Co²⁺	CoO	12.6	0.79	23.0
	Mo	Mo⁴⁺	MoO₂	11.3	5.3	25.1
	W	W⁶⁺	WO₃	13.9	1.8	10.7
	O	O²⁻	Oxides (total)	47.6	-	-
	C	C-C/C = O	Carbonates/Organics	6.1	-	-
**30 degree**	Cr	Cr³⁺	Cr₂O₃	22.2	1.8	43.9
	Co	Co²⁺	CoO	13.3	0.88	24.0
	Mo	Mo⁴⁺	MoO₂	10.7	5.7	22.0
	W	W⁶⁺	WO₃	12.7	2	10.1
	O	O²⁻	Oxides (total)	49.1	-	-
	C	C-C/C = O	Carbonates/Organics	5.6	-	-
**45 degree**	Cr	Cr³⁺	Cr₂O₃	21.5	1.9	40.9
	Co	Co²⁺	CoO	13.5	0.85	24.9
	Mo	Mo⁴⁺	MoO₂	10.9	5.6	24.9
	W	W⁶⁺	WO₃	13.2	2	9.3
	O	O²⁻	Oxides (total)	49.4	-	-
	C	C-C/C = O	Carbonates/Organics	5.4	-	-
**60 degree**	Cr	Cr³⁺	Cr₂O₃	24	2	38.2
	Co	Co²⁺	CoO	13.6	0.92	27.1
	Mo	Mo⁴⁺	MoO₂	10.5	5.8	25.2
	W	W⁶⁺	WO₃	12.8	2	9.5
	O	O²⁻	Oxides (total)	49.5	-	-
	C	C-C/C = O	Carbonates/Organics	5.2	-	-
**75 degree**	Cr	Cr³⁺	Cr₂O₃	25	2	41.3
	Co	Co²⁺	CoO	12.8	0.87	22.6
	Mo	Mo⁴⁺	MoO₂	11.1	5.6	25.4
	W	W⁶⁺	WO₃	13.1	2.1	10.8
	O	O²⁻	Oxides (total)	49.2	-	-
	C	C-C/C = O	Carbonates/Organics	5.3	-	-
**90 degree**	Cr	Cr³⁺	Cr₂O₃	25.1	2	60.2
	Co	Co²⁺	CoO	13	0.9	15.3
	Mo	Mo⁴⁺	MoO₂	10.9	5.7	12.7
	W	W⁶⁺	WO₃	12.5	2.1	11.8
	O	O²⁻	Oxides (total)	49.3	-	-
	C	C-C/C = O	Carbonates/Organics	5.4	-	-

### Microstrain analysis using the Warren-Averbach method

**Fig. 8:**
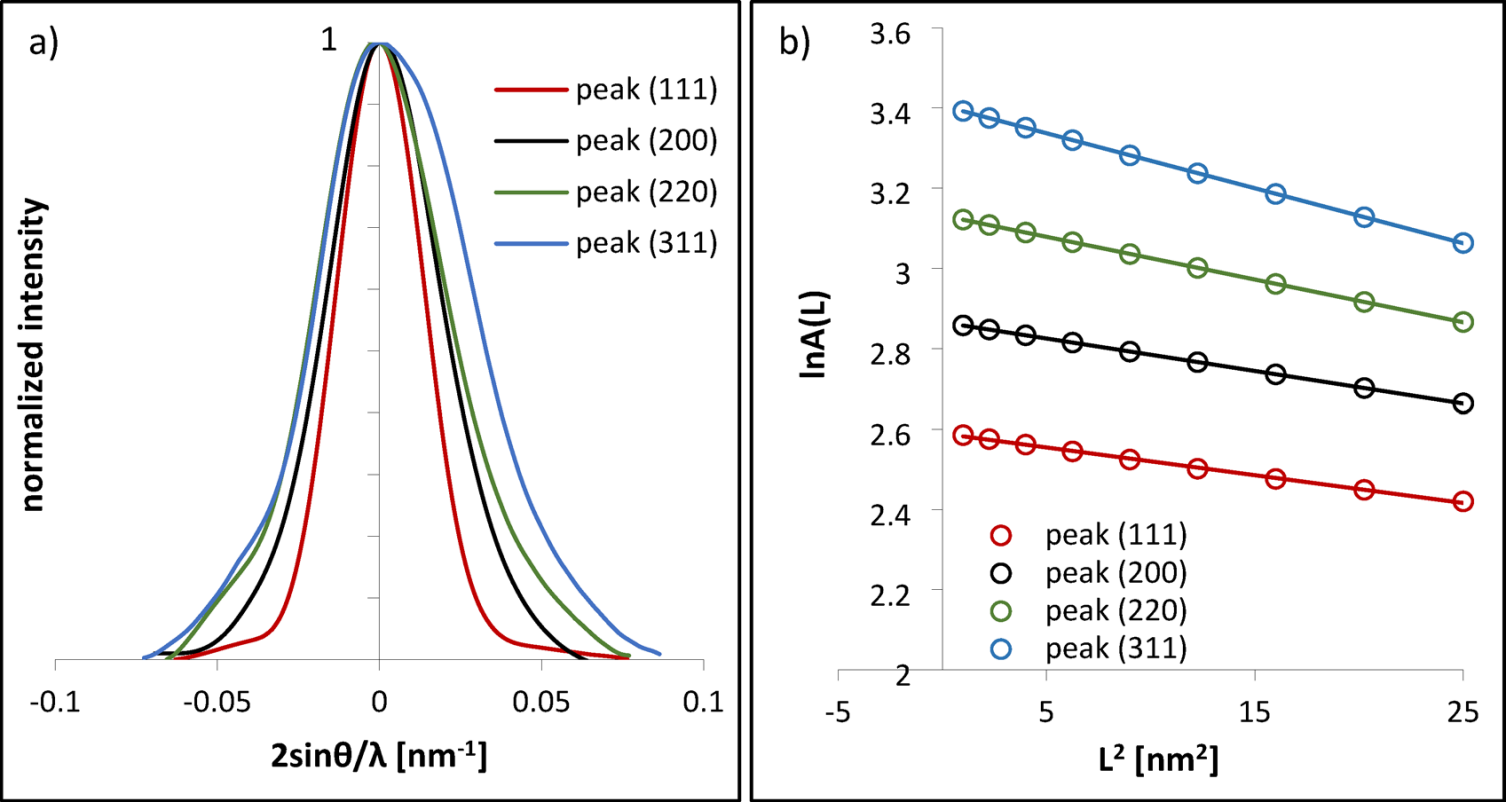
Normalized diffraction peaks for different crystallographic planes of the FCC phase in the CoCrWMo alloy, shown as a function of reciprocal space coordinate 2sinθ/λ - (**a**) and Warren-Averbach analysis: plots of lnA(L) versus L^2^ for peaks (111), (200), (220), and (311), used to assess microstrain contributions at various real-space correlation lengths - (**b**). The data correspond to the sample fabricated by L-PBF at a 90° support angle.

To assess the microstrain contribution to diffraction peak broadening, the Warren-Averbach (W-A) method was applied to four primary FCC reflections. Figure 8 shows the results for the CoCrWMo alloy fabricated at a 90° build angle. In Fig. 8a, the normalized diffraction peaks corresponding to the (111), (200), (220), and (311) planes are plotted as a function of 2sinθ/λ. After normalization and alignment, all peaks exhibit similar shapes and positions, confirming the dominance of the FCC phase and uniform crystal quality.

Figure 8b presents the dependence of the logarithm of the Fourier amplitude, lnA(L), on the square of the real-space distance, L², for each reflection. The linearity of the plots and the similarity of their slopes indicate a homogeneous distribution of microstrain across the examined lattice directions, validating the use of the W-A approach for microstrain analysis in L-PBF-processed CoCrWMo alloys.

The evolution of microstrain with build angle is shown in Fig. 8, which presents the average lnA(L) vs. L² plots for samples fabricated at different support angles (0°−90°). These curves were obtained by averaging the W-A results from four diffraction peaks. A linear relationship is evident in each case, enabling reliable estimation of microstrain. Samples printed at lower angles (0°−45°) show steeper slopes, reflecting higher microstrain, whereas flatter slopes for the 75° and 90° samples correspond to reduced lattice distortions. This trend correlates with the previously observed corrosion behavior: higher microstrain contributes to increased surface defect density, which may compromise passive film stability, while lower microstrain at higher build angles supports the formation of a more protective Cr₂O₃ layer.

Quantitative results from the Warren-Averbach analysis are summarized in Table 4. The table includes the slope of the lnA(L) vs. L² plots, average peak position g₀, microstrain (ε), and crystallite size (D) derived using the Scherrer equation. While the Cr₂O₃ passive layer on the alloy surface is only a few nanometers thick, the XRD signal predominantly reflects the bulk metallic phase due to the much greater penetration depth of X-rays. Thus, the measured microstrain corresponds to the alloy matrix rather than the oxide layer.

A notable trend is the development of crystallographic texture with increasing build angle. In particular, the (200) and (220) reflections gain intensity relative to (111) in samples printed at 75° and 90°, indicating a shift toward a < 100 > fiber texture along the build direction. This is consistent with columnar grain growth and directional solidification in vertically oriented L-PBF builds, as also observed in other Co-based alloys^5,37,38^.

Furthermore, a substantial increase in crystallite size is observed at higher support angles, with D increasing from ~ 8.5–13.0 nm (0°−60°) to 13.2 nm and 44.4 nm for 75° and 90°, respectively.

**Fig. 9:**
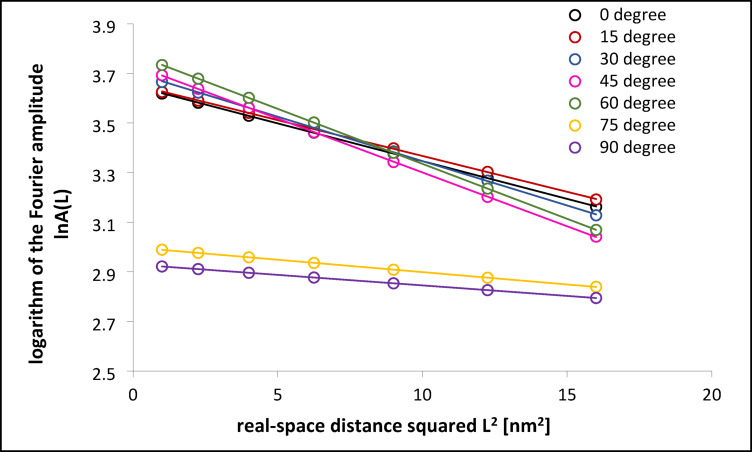
Dependence of the logarithm of the Fourier amplitude ln A(L) on the squared real-space distance L^2^ for samples built at various support angles using the L-PBF technique.

**Table 4:** Warren-Averbach results for CoCrWMo samples built at different support angles: slope of lnA(L) vs. L^2^, average peak position g_0_​, microstrain ε and crystallite size D. The crystallite size D was calculated based on the full width at half maximum (FWHM) of the (111) diffraction peak.

Built angle	Slope	g_0_ aver.	ε [-]	D [nm]
0	−0.0304	6.883	0.0057	11.4
15	−0.0289	6.881	0.0055	8.5
30	−0.0358	6.552	0.0065	10.4
45	−0.0434	6.878	0.0068	13.0
60	−0.0443	6.883	0.0069	9.3
75	−0.0098	6.882	0.0031	13.2
90	−0.0085	6.891	0.0030	44.4

This crystallite coarsening is accompanied by a significant reduction in microstrain, supporting the hypothesis that reduced defect density and larger coherent diffraction domains enhance passive film uniformity and corrosion resistance. Larger crystallites imply fewer grain and subgrain boundaries per unit volume, which are typically associated with dislocation pile-ups, segregation of alloying elements, and increased surface Energy, all of which may act as preferential sites for passive film breakdown or localized corrosion initiation. Simultaneously, lower microstrain values reflect reduced elastic lattice distortions, resulting in a more stable atomic arrangement and fewer strain-induced defects that could compromise passive layer integrity. Together, these factors favor the formation of a more continuous, adherent, and chemically stable Cr₂O₃-rich passive film. Similar effects of crystallite coarsening and reduced microstrain on passive film stability have been reported in^39^.

Therefore, the combination of lower microstrain, increased crystallite size, and preferred crystallographic orientation observed at higher support angles creates favorable microstructural conditions for the development of durable passive layers, ultimately enhancing the corrosion resistance of the CoCrWMo alloy.

### Dislocation character and density

To evaluate the dislocation character and density in L-PBF-fabricated CoCrWMo alloys, a modified Williamson-Hall (W-H) analysis was conducted using XRD data transformed into reciprocal space (see Fig. 2). Diffraction peaks corresponding to the FCC phase were fitted with Lorentzian functions to extract the full width at half maximum (ΔK) and peak center positions (K). The broadening of the (111) reflection, assumed to arise solely from crystallite size and not microstrain, was used to determine the instrumental size broadening parameter α. These values are summarized for all build angles in Table 5.

Next, linear plots of (ΔK − α)^2^/K^2^ versus H^2^, where H^2^=(h^2^k^2^ + h^2^l^2^ + k^2^l^2^)/(h^2^ + k^2^ + l^2^)^2^, were constructed for each sample (Fig. 9). The intercept of each fitted line with the H^2^ axis provided 1/q, from which the experimental dislocation character factor q was obtained.

**Table 5:** Modified Williamson-Hall analysis parameters used to evaluate crystallite size and dislocation density for different build angles. Miller indices correspond to (111), (200), and (220) reflections of the FCC CoCrWMo phase.

Built angle	Miller indices (h, k,l)	H^2^	K	ΔK	Standard error ΔK x 10^− 3^	α	KC̅ ^1/2^
**0**	(1,1,1)	0.33	4.843	0.0880	0.31	0.0792	1.69
	(2,0,0)	0	5.556	0.0971	2.06	0.0792	3.14
	(2,2,0)	0.25	7.990	0.1000	5.35	0.0792	3.30
**15**	(1,1,1)	0.33	4.830	0.1180	0.52	0.1062	1.69
	(2,0,0)	0	5.550	0.1300	4.98	0.1062	1.13
	(2,2,0)	0.25	7.880	0.1330	3.50	0.1062	3.26
**30**	(1,1,1)	0.33	4.500	0.0960	0.47	0.0864	1.64
	(2,0,0)	0	5.560	0.1070	5.53	0.0864	1.15
	(2,2,0)	0.25	7.890	0.1100	5.40	0.0864	3.22
**45**	(1,1,1)	0.33	4.840	0.0770	0.15	0.0693	1.61
	(2,0,0)	0	5.550	0.0860	1.31	0.0693	3.15
	(2,2,0)	0.25	7.880	0.0880	2.66	0.0693	3.19
**60**	(1,1,1)	0.33	4.840	0.1070	0.42	0.0963	1.60
	(2,0,0)	0	5.560	0.1200	4.88	0.0963	3.15
	(2,2,0)	0.25	7.890	0.1230	6.69	0.0963	3.18
**75**	(1,1,1)	0.33	4.840	0.0760	0.26	0.0684	1.34
	(2,0,0)	0	5.550	0.0890	0.81	0.0684	3.13
	(2,2,0)	0.25	7.880	0.0900	1.39	0.0684	2.92
**90**	(1,1,1)	0.33	4.750	0.0225	0.25	0.0203	1.23
	(2,0,0)	0	5.580	0.0275	0.89	0.0203	3.18
	(2,2,0)	0.25	7.810	0.0275	1.81	0.0203	2.83

To quantify the contributions of screw and edge dislocations, theoretical q^th^ ​ values were calculated using Eq. (8), incorporating elastic anisotropy through constants a_i_^q^, b_i_^q^, c_i_^q^ and d_i_^q^ as proposed by Ungár et al.^29^. These FCC-specific constants are listed in Table 6.

The average dislocation contrast factors C̅_h00_ for screw and edge dislocations were then determined using Eq. (13), which includes angular dependence via constants a_i_^Ch00^, b_i_^Ch00^, c_i_^Ch00^, d_i_^Ch00^ (Table 6). Based on these calculations and the experimentally determined q, the relative fractions of edge and screw dislocations were estimated by solving Eq. (7), which expresses q as a weighted combination of the two dislocation types.

**Fig. 10:**
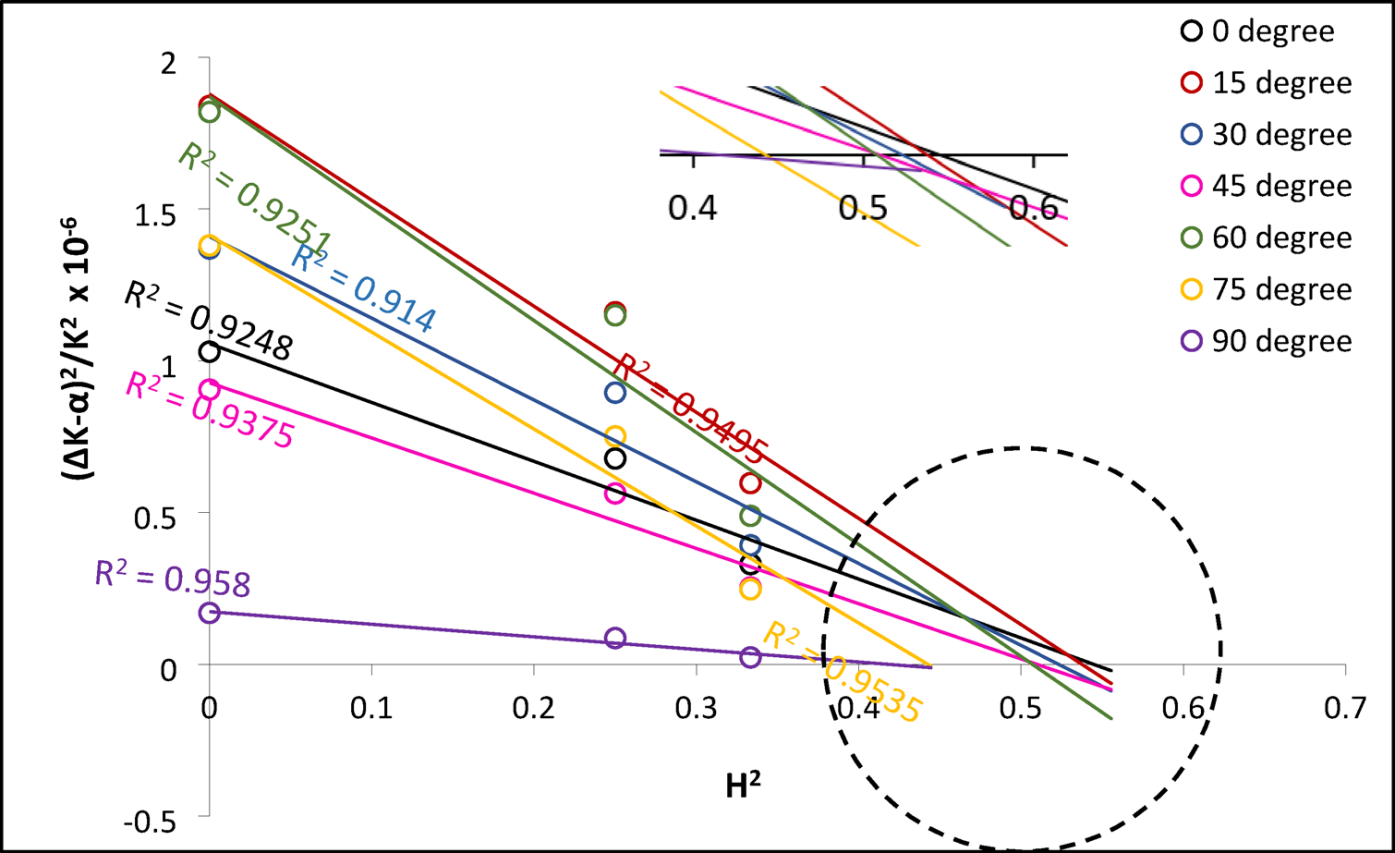
Determination of the dislocation character parameter q for CoCrWMo samples fabricated at different build angles using the modified Williamson-Hall method. The linear plots of (ΔK − α)^2^/K^2^ versus H^2^ are shown for each orientation. The inverse of the intercept with the H^2^ axis gives the value of 1/q. The quality of linear fits is indicated by the R^2^ values.

**Table 6:** Parameters needed to calculate C̅_h00_ and *q*_*i*_^*th*^ for CoCrWMo in Fcc crystal.

	Parameter q_i_th^29^				factor C̅_h00_^29^			
	a^q^_i_	b^q^_i_	c^q^_i_	d^q^_i_	a_i_^Ch00^	b_i_^Ch00^	c_i_^Ch00^	d_i_^Ch00^
**Screw**	5.4252	0.7196	0.069	−3.1917	0.174	1.9522	0.0293	0.0662
**Edge**	4.8608	0.8687	0.0896	−3.428	0.1687	2.04	0.0194	0.0926

Once the dislocation character was established, the average dislocation contrast factor C̅_h00_ for each sample was computed as a weighted mean of the values for edge and screw dislocations. These, along with the individual and average C̅_h00_​, experimental and theoretical q, and dislocation fractions, are presented in Table 7.

**Fig. 11:**
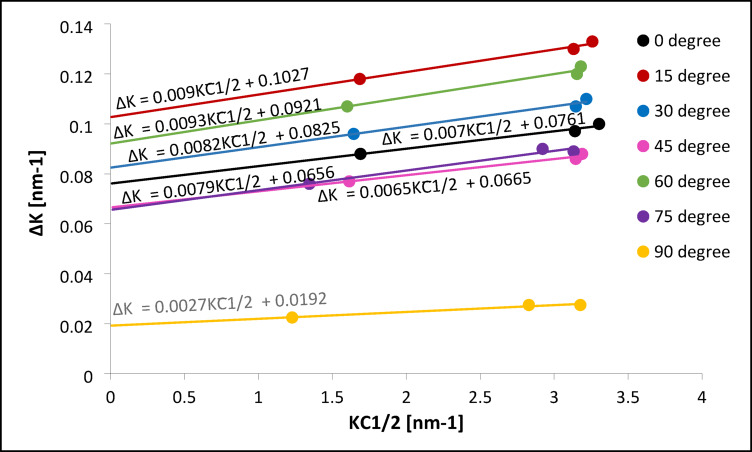
Modified Williamson-Hall plots of peak broadening ΔK as a function of K· C̅ ^1/2^ for CoCrWMo alloy samples fabricated by L-PBF at different support angles (0°−90°). The slope of each line is proportional to the square root of dislocation density.

The overall contrast factor C̅ and the product KC̅ ^1/2^ were then calculated (Eq. 11), and values for each reflection and build orientation are listed in Table 5. Using these, plots of ΔK versus KC̅ ^1/2^ were generated and linearly fitted (Fig. 11). The intercept of each fit was used to calculate the crystallite size (via the Scherrer term), while the slope provided the dislocation density, using Eq. (11). These final values are summarized in Table 8.

The results reveal a clear correlation between dislocation structure and corrosion resistance. The sample built at 90° showed the lowest dislocation density (0.94 × 10¹³ m⁻²) and the largest crystallite size (46.88 nm), indicating fewer lattice defects acting as corrosion initiation sites. Additionally, this sample had the highest proportion of screw dislocations (67.5%), which are known to induce less surface lattice distortion than edge dislocations.

**Table 7:** Theoretical value of q^th^ for screw and edge dislocations, the fraction of screw and edge dislocations, theoretical value of dislocation contrast for screw and edge dislocations, the average dislocation contrast factor C̅_h00_ for the (h00) reflections for all the tested samples.

built angle	Q	q^th^_screw_	q^th^_edge_	f^edge^	f^screw^	C̅_h00screw_	C̅_h00edge_	C̅_h00_
**0**	1.852	2.490	1.744	0.855	0.145	0.335	0.315	0.318
**15**	1.859	2.490	1.744	0.855	0.145	0.335	0.315	0.318
**30**	1.923	2.490	1.744	0.760	0.240	0.335	0.315	0.320
**45**	1.961	2.490	1.744	0.709	0.291	0.335	0.315	0.321
**60**	1.980	2.490	1.744	0.683	0.317	0.335	0.315	0.322
**75**	2.128	2.490	1.744	0.562	0.438	0.335	0.315	0.324
**90**	2.247	2.490	1.744	0.325	0.675	0.335	0.315	0.329

In contrast, samples fabricated at lower build angles (e.g., 15° and 30°) exhibited significantly higher dislocation densities (10.5 and 8.71 × 10¹³ m⁻²), smaller crystallite sizes (8.76 and 10.91 nm), and lower screw dislocation fractions (14.5% and 24.0%). The predominance of edge dislocations in these samples increases lattice strain and local energy near the surface, making them more prone to passive film breakdown and localized corrosion.

**Table 8:** Linear fit parameters obtained from plots of ΔK versus K· C̅ ^1/2^ for CoCrWMo samples fabricated at different build angles.

Built angle	Slope	ΔK -intercept	D* [nm]	ρ x10^13^ [m^− 2^]
**0**	0.0070	0.0761	11.83	6.35
**15**	0.0090	0.1027	8.76	10.5
**30**	0.0082	0.0825	10.91	8.71
**45**	0.0065	0.0665	13.74	5.47
**60**	0.0093	0.0921	9.77	11.2
**75**	0.0079	0.0656	13.72	8.08
**90**	0.0027	0.0192	46.88	0.94

These findings demonstrate that microstructures characterized by a higher fraction of screw dislocations, larger crystallites, and lower dislocation densities are associated with improved corrosion resistance. This agrees with the XPS results, which indicated a higher relative Cr₂O₃ content in the passive layer of the 90° sample, supporting its improved corrosion resistance and microstructural characteristics. Mechanistically, dislocation structure can influence passive film formation in Co-based alloys. While moderate densities of edge dislocations may promote chromium diffusion and accelerate initial passivation, excessive dislocation content, especially near the Surface, can introduce high-energy sites that compromise passive film integrity. Screw dislocations, with their lower associated strain fields, may reduce such effects and contribute to more stable passivation. Lower dislocation densities, particularly at higher build angles, result in more structurally ordered subsurface regions that support the development of continuous and adherent Cr₂O₃ layers.

These results are consistent with prior reports showing that dislocation character and density impact the corrosion behavior of additively manufactured metals^40–42^. Therefore, optimizing the build orientation in L-PBF not only governs mechanical performance but also critically affects the electrochemical stability of CoCrWMo components.

This highlights the importance of controlling dislocation structure-through processing conditions such as build angle-to tailor both the mechanical and corrosion performance of CoCrWMo biomedical alloys.

## Conclusion

This study demonstrates that build orientation in the L-PBF process has a pronounced influence on the microstructure, dislocation character, and corrosion behavior of CoCrWMo alloys. Samples fabricated at a 90° build angle exhibited the highest corrosion resistance, which correlated with the lowest dislocation density, the highest fraction of screw dislocations, and the lowest microstrain levels, as determined by modified Williamson-Hall and Warren-Averbach analyses.

The superior corrosion performance of these samples is attributed to reduced internal strain energy and the formation of a more stable passive film, enriched in Cr and W oxides. In contrast, samples printed at lower support angles showed elevated dislocation densities, higher fractions of edge dislocations, and increased microstrain, factors that collectively promote local strain accumulation, passive film instability, and greater susceptibility to electrochemical degradation.

These findings highlight the critical role of crystallographic anisotropy and defect structure, particularly dislocation type and density, in governing the corrosion response of L-PBF-fabricated Co-based alloys. Tailoring build orientation thus emerges as a viable strategy for optimizing microstructure-sensitive properties such as corrosion resistance, especially in biomedical or chemically aggressive environments.

The results support a design approach based on process parameter optimization to improve corrosion resistance without the need for additional protective coatings or alloying elements, promoting more sustainable and material-efficient production of both biomedical and engineering components.

Moreover the electrochemical response observed in this study indicates that CoCrMo alloys predominantly undergo transpassive dissolution rather than true pitting corrosion within the investigated potential range, consistent with previously reported behavior for this alloy system.

Future work should integrate EBSD, TEM, and XPS depth profiling to better elucidate the interplay between local deformation fields, passive layer composition and electrochemical behavior, ultimately guiding the design of functionally durable additively manufactured components.

## Supplementary Information

Below is the link to the electronic supplementary material.

Supplementary Material 1

## Data Availability

The data that support the findings of this study are available from the corresponding author upon reasonable request.
