# Supplementary material for: Influence of laser powder bed fusion build orientation on the corrosion resistance of CoCrWMo alloy considering dislocation density character and lattice microstrain
Source: Sci Rep. 2025 Nov 28;15:45625. doi: 10.1038/s41598-025-30143-w (PMC12753813; doi:10.1038/s41598-025-30143-w)
Supplement: Supplementary file 1 — Supplementary Material 1 [file 41598_2025_30143_MOESM1_ESM.docx]

Supplementary Information Figure S1


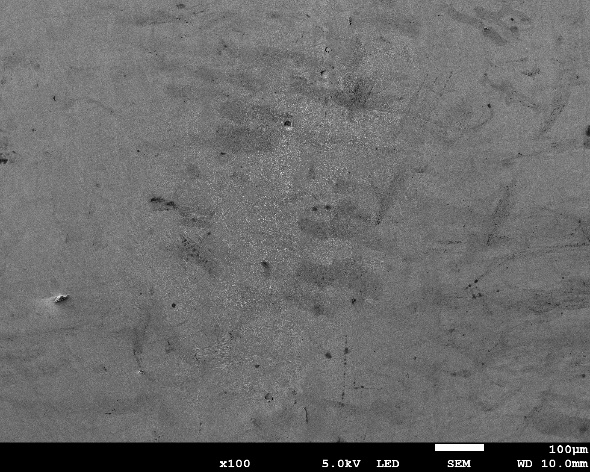

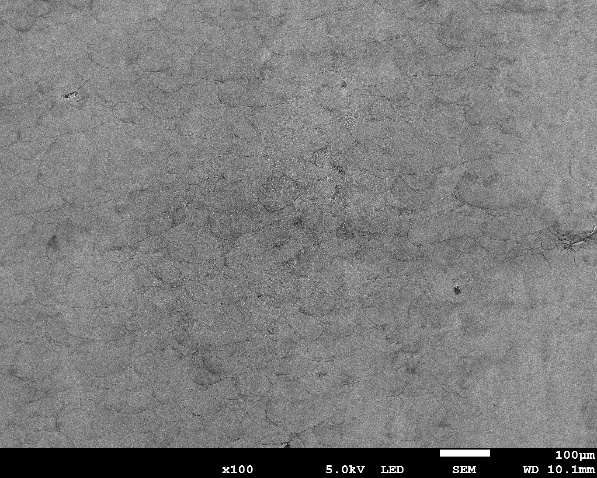


b)

a)


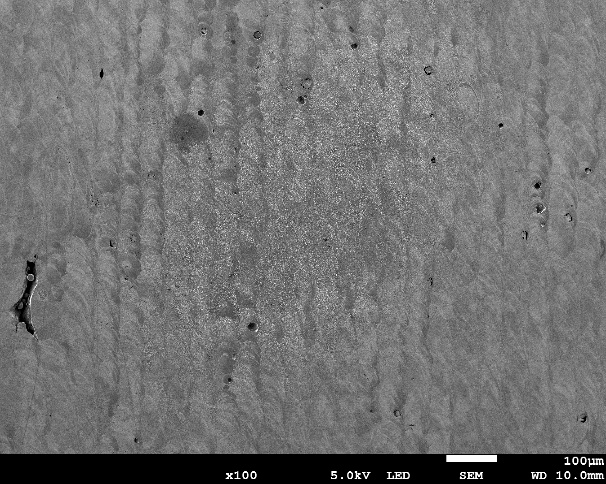

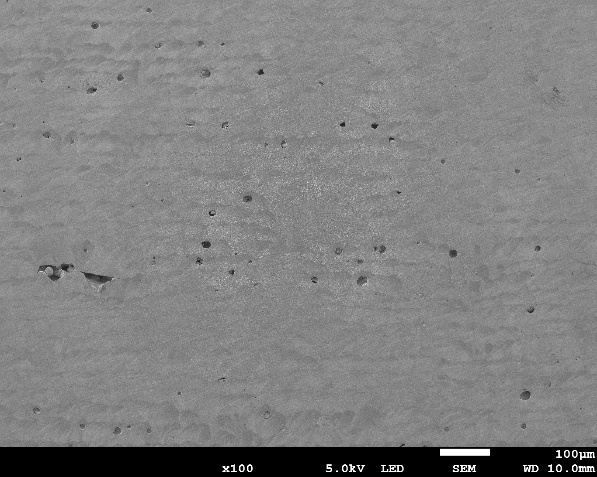


d)

c)


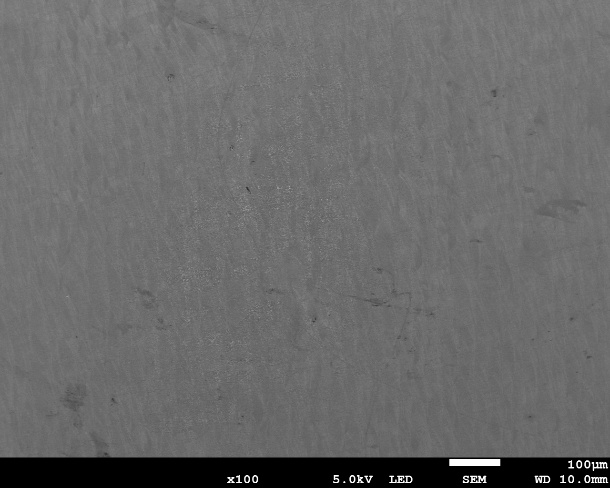

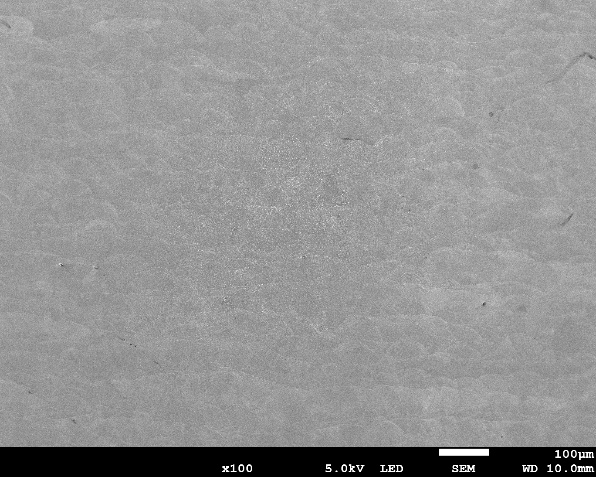


f)

e)


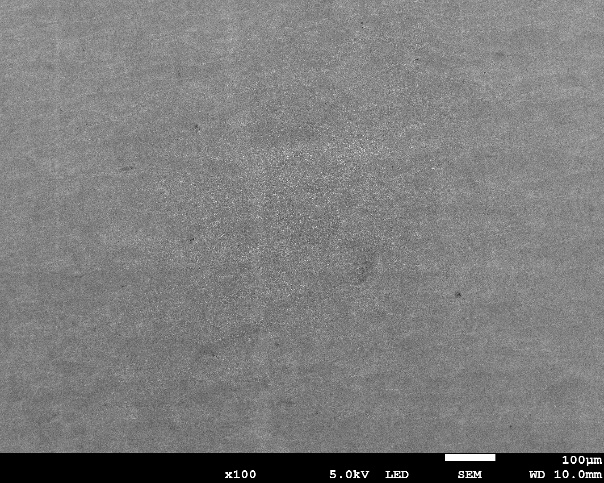


g)

Fig. S1. SEM micrographs of the surfaces of CoCrWMo samples before electrochemical corrosion tests, printed at different build angles:
a) 0°, b) 15°, c) 30°, d) 45°, e) 60°, f) 75°, and g) 90°.
